# Role of bilateral staged hip arthroplasty in Hip-spine syndrome: A case report

**DOI:** 10.1097/MD.0000000000036296

**Published:** 2023-12-08

**Authors:** Aren Joe Bizdikian, Ayman Assi, Karl Semaan, Joeffroy Otayek, Mohamad Karam, Abir Massaad, Elena Jaber, Ismat Ghanem, Rami El Rachkidi

**Affiliations:** a Laboratory of Biomechanics and Medical Imaging, Faculty of Medicine, Saint-Joseph of Beirut, Beirut, Lebanon.

**Keywords:** adult spinal deformity, case report, gait analysis, hip osteoarthritis, hip-spine syndrome

## Abstract

**Rationale:**

Hip-spine syndrome is a frequent finding in patients presenting with symptoms both at the level of the hip and spine.

**Patient concerns:**

Patient previously operated of lumbar laminectomy for supposed spinal stenosis presenting with persistent pain and disability.

**Diagnoses:**

Clinical examination and imaging showed severe bilateral hip osteoarthritis. Full body standing and sitting biplanar radiographs showed an associated severe sagittal malalignment. 3D motion analysis and health-related quality of life (HRQOL) questionnaires showed a severe functional impact.

**Interventions:**

He was operated of a staged bilateral total hip arthroplasty using the direct anterior approach.

**Outcomes:**

Spinopelvic and sagittal alignment parameters, as well as 3D motion analysis and HRQOL scores showed significant improvement after the first, then the second total hip arthroplasty.

**Lessons:**

Comprehensive functional diagnostic testing, including full body standing and seated radiographs, 3D gait analysis and HRQOL questionnaires may provide important information for future management.

## 1. Introduction

The interrelation between hip and spine pathologies is an ever-evolving concept. In fact, hip-spine syndrome was first described in 1983 by Offierski and MacNab.^[1]^ Even in patients with a healthy spine, hip pathologies are known to alter the pelvic orientation thereby affecting the lumbar spine and leading to low-back pain.^[2]^ Conversely, spinal pathologies may also lead to uneven stresses on the hips. This is especially due to acetabular anterior under-coverage from pelvic retroversion leading to early degenerative changes.^[3,4]^ This close relationship between hip and spine pathologies often leads to confusion during diagnosis, with the differentiation between the origin of the symptoms challenging to make.^[5]^ One example is the fact that lumbar spinal stenosis and hip osteoarthritis (OA) may present with similar symptoms.^[6]^

While simple cases are easily distinguished, complex hip-spine syndrome requires extensive evaluation for proper diagnosis. If inappropriate treatment is then undertaken, the hip-spine syndrome is deemed misdiagnosed.^[1,5]^ Moreover, the interplay between the 2 entities renders physical examination difficult to undertake and often requires therapeutic tests such as injections to determine the cause of the symptoms.

Increasing importance is being given to the functional assessment of spinal deformities. The most readily available tool for such an assessment being 3D movement analysis, the dynamic concept of postural assessment has been brought to the foreground by many authors.^[7]^ As such, the functional assessment of subjects with adult spinal deformity during walking^[8,9]^ and other activities of daily living, such as sitting and standing^[10,11]^ has been recently reported, further highlighting the importance of such an assessment in the complete evaluation of patients with hip and spine pathologies.

We present the case of a patient with a misdiagnosed hip-spine syndrome due to hip OA evaluated both in a static manner using radiographs and 3D movement analysis to assess the functional impact.

## 2. Case report

This is the case of a 52-year-old male patient, weighing 131 Kg and measuring 186 cm, presenting with a long history of low back pain along with bilateral hip and thigh pain. He reports an increase in pain intensity in the last few years which has become refractory to conservative management.

Ten years before presentation, an L4-5 lumbar laminectomy had been performed at a different center for supposed lumbar spinal stenosis with no improvement of his symptoms. Actually, the patient presented for a second opinion after being told he required extension of the lumbar laminectomy proximally with instrumentation and fusion due to refractory pain.

On physical exam, a severe sagittal imbalance was found on physical exam with the spine shifted anteriorly, the pelvis anteverted, and the knees flexed. A painful gait was also noted. Motor strength was normal for all lower extremity muscle groups bilaterally with a score of 5/5, sensitivity was normal and symmetrical, Achilles and patellar reflexes were 2 + bilaterally and plantar reflexes were normal bilaterally. The remainder of the neurovascular exam extremities was normal for both lower limbs. Examination of the hips revealed a 5° flexion deformity bilaterally. Range of motion (ROM) was limited in internal and external rotation and abduction bilaterally (Right: extension/flexion 0°/0°/60°, internal/external rotation: 0°/0°/10°; abduction/adduction 15°/0°/10°. Left: extension/flexion 0°/0°/70°, internal/external rotation: 0°/0°/10°, abduction/adduction: 10°/0°/10°) with pain on mobilization. Positive flexion adduction internal rotation and flexion abduction external rotation tests were found bilaterally.

**Figure 1.**
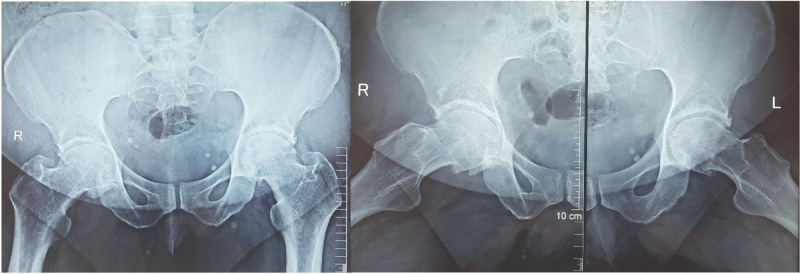
Anteroposterior radiographs of the pelvis and anteroposterior and lateral radiographs of bilateral hips at baseline showing severe bilateral hip osteoarthritis.

Conventional radiographs of the pelvis and both hips were performed and showed severe hip OA Tonnis grade 3 with coxa profunda bilaterally (Fig. 1).

The patient was then referred to our center to benefit from further extensive diagnostic evaluation and functional assessment, in the setting of an ongoing research project (IRB: CEHDF1259). All subjects included in the study have signed an informed consent form for the anonymous use of their data for scientific purposes.

**Figure 2.**
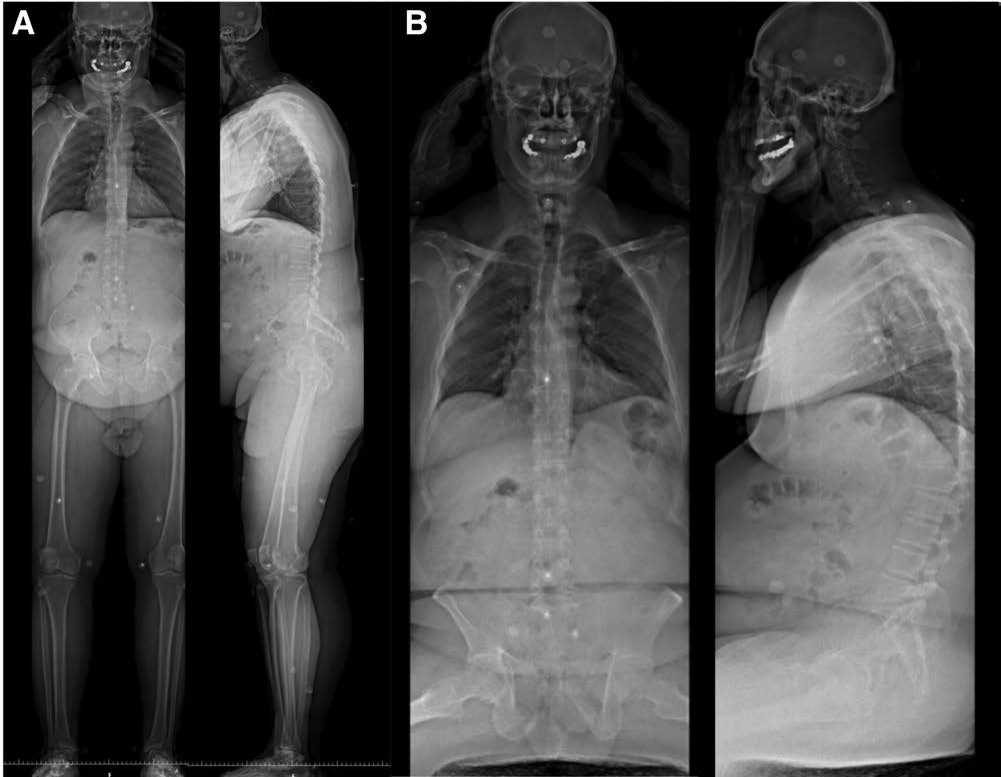
Baseline standing (A) and seated (B) full-body biplanar radiographs.

To assess the postural alignment of the patient, full body standing biplanar radiographs (EOS^®^ Imaging, Paris, France) were acquired. The patient was instructed to stand in the standardized free-standing position.^[12,13]^ The radiographs were then repeated with the patient in the seated position on a height-adjustable chair without a backrest, with the knees and hips bent at 90°.^[14]^ The standing and seated radiographs are presented in Figure 2.

Three-dimensional reconstructions of the spine and the lower extremities were performed using the SterEOS^®^ system (EOS^®^ Imaging) and the pelvis using a specific software (Arts et Métiers ParisTech, Paris, France). The following sagittal spinopelvic alignment parameters were generated: pelvic incidence (PI, in°), pelvic tilt (PT, in°), sacral slope (SS, in°), L1S1 lumbar lordosis (LL, in°), L4S1 lordosis (L4S1, in °), PI-LL mismatch (PI-LL, in°), and T1T12 thoracic kyphosis (TK, in°).

Global postural alignment parameters were also calculated, including the sagittal vertical axis (SVA, in mm), odontoid to center to hip axis (ODHA, in °; with −2 to 2° considered within the normal range) and knee flexion of the right lower limb (KF, in°).

The patient underwent 3D gait analysis using 8 optoelectronic cameras (Vicon^®^, Oxford, UK). The Davis and Leardini protocols were utilized for the placement of 41 reflecting markers over bony landmarks at the level of the lower limbs and trunks.^[15,16]^ The patient was asked to walk barefoot at a self-selected speed on a 10-m walkway several times. Time-distance parameters and kinematic waveforms were obtained during the gait cycle of the trunk, spine, pelvis, and lower limb joints in 3D using Nexus® and Procalc® (Vicon^®^).

Finally, the patient filled out the following health-related quality of life (HRQOL) questionnaires: Oswestry Disability Index (ODI), 36-Item Short-Form Health Survey (SF-36) with the physical and mental component summaries (PCS and MCS, respectively), Visual Analog Score (VAS) for pain, and Beck’s Depression Inventory (BDI).

### 2.1. Results at baseline

The patient had a PI = 43°, PT = −2°, LL = 36°, PI-LL = 7°, TK = 38°, PT = −2°, SVA = 110°, and ODHA = 13° in the standing position. In the seated position, the patient had a PT = 33°, LL = 11°, PI-LL = 29°, TK = 38°, PT = 33°, and SVA = 60° (Table 1, Fig. 2).

**Table 1:** Global alignment and spinopelvic parameters at baseline, following right and left total hip arthroplasty (RTHA and LTHA, respectively) in both standing and seated positions.

	Standing position				Seated position		
	Baseline	After RTHA	After LTHA	Baseline		After RTHA	After LTHA
SVA (mm)	110	57	18	60		86	34
ODHA (°)	13	5	1	1		6	4
KF (°)	16	3	3				
LL (°)	36	42	50	11		35	32
TK (°)	38	37	40	38		39	33
PI-LL (°)	7	1	−5	29		10	11
PI (°)	43	43	43	43		43	43
SS (°)	44	41	37	10		30	18
PT (°)	−2	2	6	33		13	25

KF = knee flexion of the right lower limb, LL = lumbar lordosis, ODHA = odontoid to center of hip axis, PI = pelvic incidence, PT = pelvic tilt, SS = sacral slope, SVA = sagittal vertical axis, TK = thoracic kyphosis.

**Figure 3.**
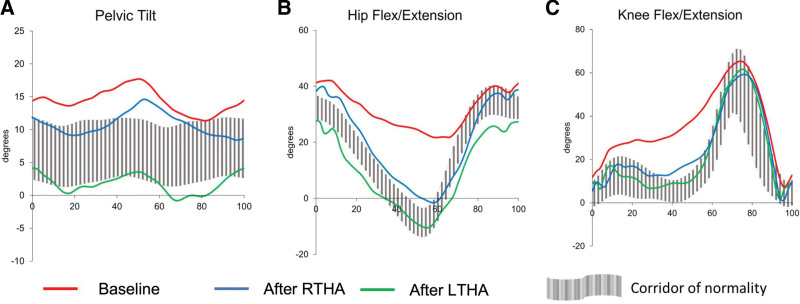
Pelvic tilt (A), hip flexion/extension (B), and knee flexion/extension (C) during the normalized gait cycle as calculated using 3D gait analysis of both lower limbs at baseline, and after right and left total hip arthroplasty (RTHA and LTHA, respectively).

Gait analysis data showed an anteverted pelvis, persistent hip flexion with reduced ROM bilaterally, as well as a persistent knee flexion with reduced ROM bilaterally throughout the gait cycle. While all time-distance variables were within normal values, they were mostly at the limits with asymmetry between the right and left lower limbs in terms of step length (0.62 m vs 0.54 m, respectively) (Table 2, Fig. 3).

**Table 2:** Time-distance gait analysis parameters at baseline and following right and left total hip arthroplasty (RTHA and LTHA, respectively).

	Normal range	Right lower limb			Left lower limb		
		Baseline	After RTHA	After LTHA	Baseline	After RTHA	After LTHA
Walking speed (m/s)	1–1.2	0.93	1.09	1.15	0.94	1.11	1.18
Cadence (steps/min)	98.3–111.7	96.8	101	102.3	96.8	103	103.5
Foot off (%)	56.3–61.1	66.9	66.4	63.1	62.9	62.4	62.9
Single support (s)	0.3–0.5	0.46	0.44	0.44	0.41	0.39	0.43
Step length (m)	0.5–0.7	0.62	0.62	0.68	0.54	0.68	0.69
Double support (s)	0.1–0.3	0.32	0.35	0.3	0.37	0.34	0.3

QOL scores showed a SF-36 PCS and MCS components scores of 39.2 and 57.4, respectively, ODI of 40, BDI of 7 and VAS of 8 (Table 3).

**Table 3:** PROMs at baseline and following right and left total hip arthroplasty (RTHA and LTHA, respectively).

		Baseline	After RTHA	After LTHA
SF36	PCS	39.2	39.9	49.5
	MCS	57.4	59.5	60.3
ODI		40	18	8
BDI		7	5	5
VAS		8	7	1

BDI = Beck Depression Inventory, MCS = Mental Component Summary, ODI = Oswestry Disability Index, PCS = Physical Component Summary, PROMs = patient reported outcomes measures, SF-36 = 36-Item Short-Form Health Survey, VAS = Visual Analog Score.

### 2.2. Diagnosis and treatment plan

Based on the above data, the diagnosis of severe hip OA with associated hip-spine syndrome was made. The patient was ultimately operated of a staged bilateral total hip arthroplasty (THA) using a direct anterior approach, starting with the right side. The interval between the surgeries was set at 3 months.

All of the above-cited investigations were undertaken twice more, 1 month after the right THA and again after the left side to evaluate the effect of a staged bilateral hip replacement on the patient’s sagittal alignment.

### 2.3. Results after right THA

**Figure 4.**
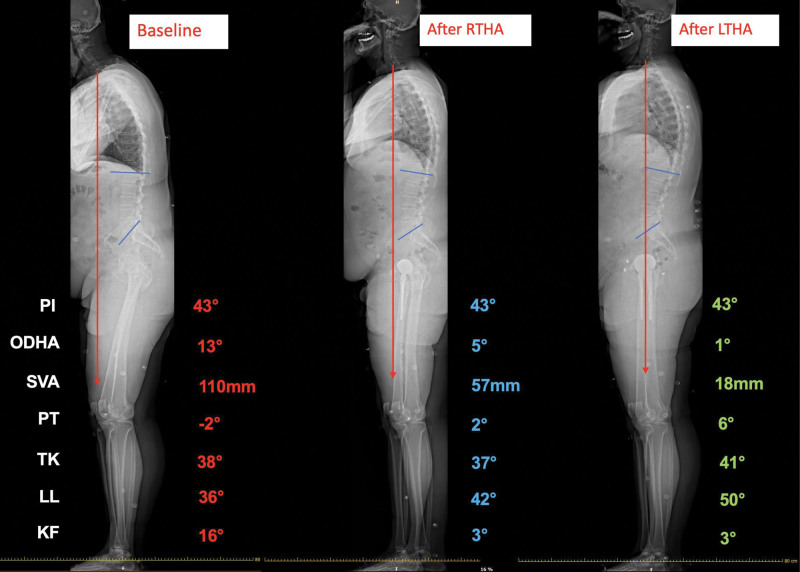
Full body sagittal spinal radiographs showing the evolution of global alignment and spinopelvic parameters at baseline and following right and left total hip arthroplasty (RTHA and LTHA, respectively). CAM-HA = center of the auditory meatus to hip axis, KF = knee flexion, LL = lumbar lordosis, PI = pelvic incidence, PT = pelvic tilt, SVA = sagittal vertical axis, TK = thoracic kyphosis.

Global alignment parameters showed a decrease in SVA to 57mm, ODHA to 5° and KF to 3° and an increase in LL to 42° in the standing position, while PT slightly increased to 2°. In the seated position, LL increased to 35°, PI-LL decreased to 10° and PT to 13° (Table 1, Fig. 4).

Time-distance parameters showed a slight increase in both walking speed (1.09 and 1.11 m/s, respectively), cadence (101 and 103 steps/min, respectively), and step length (0.62 and 0.68 m, respectively) on the right and left lower limbs, respectively. Furthermore, gait kinematic parameters showed a slight decrease in pelvic tilt, a notable decrease in hip flexion and knee flexion during the entire gait cycle with a wider, more normal ROM (Table 2, Fig. 3).

Health-related quality of life measures also showed an improvement, with a decrease in ODI to 18 and BDI to 5. Nevertheless, VAS and SF-36 did not show any remarkable improvements (Table 3).

### 2.4. Results after left THA

Global alignment parameters showed a further decrease in SVA to 18mm and ODHA to 1°, a further increase in LL to 50°, while KF remained 3° in the standing position. PT increased to 6°. In the seated position, LL and PI-LL remained virtually unchanged, while PT increased slightly to 25° (Table 1, Fig. 4).

Time-distance parameters showed a further increase in both walking speed (1.15 and 1.18 m/s, respectively), cadence (102.3 and 103.5 steps/min, respectively), and step length (0.68 vs 0.68, respectively) which also became more symmetrical, on the right and left lower limbs, respectively. Furthermore, gait kinematic parameters showed a further decrease in pelvic tilt, a further decrease in hip flexion and knee flexion during the entire gait cycle with a wider, more normal ROM (Table 2, Fig. 3).

Finally, HRQOL measures showed further improvement, with an increase in the SF-36 PCS to 49.5, and a decrease in ODI to 8, BDI to 5 and VAS to 1 (Table 3).

## 3. Discussion

Assessment of the entire spinal alignment with the pelvis and hips has provided much insight into the interrelation between the hip and spine, with increasing importance being given to hip-spine and spine-hip syndromes. Moreover, the ability to reconstruct the spine in 3D based on radiographs, as is available with biplanar X-rays, has further advanced our understanding of these pathologies in an unprecedented manner. When radiographic evaluation is coupled with 3D motion analysis, both the static and dynamic impact of hip OA may be evaluated as a whole. As such, alterations in gait and the radiographic impact of degenerative processes on the pelvis and the spine may be highlighted. Some studies even suggest that gait analysis be a routine part of pre-operative hip OA assessment.^[17]^ In this report, we present a case of a misdiagnosed hip-spine syndrome where a lumbar laminectomy did not provide pain relief. Hip OA was subsequently diagnosed, and a staged bilateral total hip arthroplasty improved the patient’s sagittal alignment, gait and quality of life.

The patient’s pre-operative sagittal alignment is similar to that described in the literature for patients with hip OA, notably a low standing PT with increased hip flexion and a high SVA.^[2,18,19]^ According to the Hip-spine syndrome classification published by Diebo et al^[20]^ in 2019, our patient would fall under the hip-type. This type includes patients with grade 3 to 4 hip OA and a PI-LL ≤ 10°.

This patient presented with alterations at the pelvic level that are typically seen in patients with hip OA. This includes anterior PT,^[21,22]^ as is evident by a high SS. According to the SS = PI*0.54 + 12 and PT = 0.44*PI-11.5 formulas,^[23]^ the theoretical SS and PT in a patient with a PI = 43° should be around 35° and 7°, compared to 44° and −2° in this patient, respectively. This pelvic anteversion is, according to Diebo’s classification,^[20]^ a hallmark of hip OA without spinal malalignment (PT is often < 15° in this subgroup) and is typically due to hip flexion contractures.^[24]^

Furthermore, this patient presented with a healthy lumbo-pelvic complex, and although SVA and ODHA were high at baseline (110mm and 13°, respectively), their PI-LL remained within the normal range. A healthy lumbo-pelvic complex was further evident by a change in SS larger than 20° between the standing and seated positions.^[14]^ In fact, the seated position generally forces the pelvis to rotate posteriorly to allow proper flexion of the hips without causing anterior femoro-acetabular impingement, which secondarily causes changes in LL as a response to the decrease in SS.^[25]^

The patient’s spinal sagittal balance progressively improved after the right and then the left total hip arthroplasty. In fact, the SVA (110 mm vs 57 mm vs 18 mm, respectively) and ODHA (13° vs 5° vs 1°, respectively) showed marked improvement after the staged procedure. Moreover, the abnormal pelvic anteversion found in this patient returned to its theoretical baseline value at final follow up (PT = −2° vs 2° vs 6°, respectively). Although PI-LL was within 10° pre-operatively, L4S1 was 30° for an L1S1 of 36° (84% of L1LS1) at baseline, compared to 32° for an L1S1 of 50° (68% of L1LS1) at final follow-up. This shows a more harmonious shape of the spine at final follow-up compared to the pre-operative state. Pelvic mobility also improved remarkably with a return to normal retroversion in the seated position (SS = 10° vs 30° vs 18°), similarly to what is found in the literature.^[26,27]^

Knee flexion is often seen as a characteristic of adult spinal deformity: the pelvis goes into posterior tilt in an attempt to compensate for decreased LL. When maximal PT is reached, progressive spinal degeneration requires further compensation. This leads to posterior femoro-acetabular impingement, thus driving flexion at the level of the knees in order to further retrovert the pelvis.^[23,28]^ In our patient, a knee flexion of 16° may be misleading toward the diagnosis of ASD. However, since the pelvis is in anteversion in this patient, knee flexion is rather due to hip flexion contracture from hip OA.

Gait kinematic data also showed gradual improvement in the patient’s walking ability. The anteriorly tilted pelvis gradually became more retroverted, hip ROM increased and was no longer in flexion, and knee ROM increased and was no longer in flexion. These findings are similar to those found in the literature.^[17]^

Walking speed, cadence and step length also improved postoperatively. Interestingly, after THA on the right side only, the left side also showed improvement in time-distance parameters, especially step length. Both sides further benefitted after both hips were operated of THA. This was most evident with step length, which was asymmetrical between both lower limbs at baseline, and while the left side improved after right sided THA, symmetry was achieved only after both sides were operated. This shows how a pathological process involving 1 hip can affect the contralateral hip, and by simply treating 1 side, the other side would automatically show objective improvement.

While the above findings are objective measures used to assess patient outcomes, patient-related outcome measures remain at the forefront of measuring treatment success. In our study, we opted to assess 3 general areas of the patient’s quality of life, including pain using the VAS, disability with the ODI (a tool specifically designed for the assessment of functional decline and disability due to lower back pain,^[29]^ and general health using the SF-36 (a comprehensive score encompassing both physical and mental health components). Pain significantly decreased in this patient between the pre-operative, first and second staged surgeries (VAS = 8 vs 7 vs 1, respectively). In addition, the patient reported lower ODI scores between the different assessments. While the original ODI score was 40, indicating severe disability, successive decreases of the score to 18 and finally to 8 show a tremendous gain in daily quality of life and thus function by the patient. Moreover, the SF-36, increased after the staged procedure, showing improvements in both physical ability and the patient’s mental status. Compared to the radiographic and motion analysis parameters, HRQOL did not substantially improve until the final follow-up, and thus until both hips were operated on. This shows the significant impact unilateral hip OA can have on the patient’s daily activities.

## 4. Conclusion

In conclusion, the relationship between the hip and spine are closely intertwined. Disease in one of these 2 invariably leads to alterations in the other. This case illustrates the importance of adequately diagnosing patients presenting with symptoms either at the level of the hip, the spine or both. Comprehensive functional diagnostic testing, including full body standing and seated radiographs, along with 3D gait analysis and HRQOL questionnaires, may provide important information and should be considered in patients with hip-spine or spine-hip syndromes. Furthermore, when considering staged hip replacement procedures, repeating the complementary examinations at each stage may provide valuable insight with the patient’s progress, especially in complicated cases of hip-spine syndrome.

## Author contributions

**Conceptualization:** Ayman Assi, Abir Massaad, Rami El Rachkidi.

**Data curation:** Karl Semaan, Mohamad Karam, Elena Jaber.

**Formal analysis:** Aren Joe Bizdikian, Joeffroy Otayek, Abir Massaad, Rami El Rachkidi.

**Funding acquisition:** Ayman Assi, Ismat Ghanem.

**Investigation:** Karl Semaan, Mohamad Karam, Elena Jaber, Rami El Rachkidi.

**Methodology:** Ismat Ghanem, Rami El Rachkidi.

**Project administration:** Ayman Assi, Ismat Ghanem.

**Resources:** Ayman Assi.

**Supervision:** Ayman Assi, Mohamad Karam, Abir Massaad, Rami El Rachkidi.

**Validation:** Ismat Ghanem, Rami El Rachkidi.

**Writing – original draft:** Aren Joe Bizdikian.

**Writing – review & editing:** Aren Joe Bizdikian, Ayman Assi, Joeffroy Otayek, Rami El Rachkidi.
